# Germline *BRCA1/2* status and chemotherapy response score in high-grade serous ovarian cancer

**DOI:** 10.1038/s41416-024-02874-6

**Published:** 2024-11-16

**Authors:** Robert D. Morgan, Xin Wang, Bethany M. Barnes, Laura Spurgeon, Aurore Carrot, Daniel Netto, Jurjees Hasan, Claire Mitchell, Zena Salih, Sudha Desai, Joseph Shaw, Brett Winter-Roach, Helene Schlecht, George J. Burghel, Andrew R. Clamp, Richard J. Edmondson, Benoit You, D. Gareth R. Evans, Gordon C. Jayson, Stephen S. Taylor

**Affiliations:** 1 Department of Medical Oncology, The Christie NHS Foundation Trust, Manchester, United Kingdom; 2 Division of Cancer Sciences, School of Medical Sciences, Faculty of Biology, Medicine and Health, The University of Manchester, Manchester, United Kingdom; 3 Manchester Academic Health Science Centre (MAHSC), Manchester, United Kingdom; 4 Clinical Outcome and Data Unit, The Christie NHS Foundation Trust, Manchester, United Kingdom; 5 Centre pour l’lnnovation en Cancérologie de Lyon (CICLY), EA 3738, Université Claude Bernard University Lyon 1, Univ Lyon 1, Oullins-Pierre-Bénite, France; 6 Department of Histopathology, The Christie NHS Foundation Trust, Manchester, United Kingdom; 7 Department of Histopathology, Manchester University NHS Foundation Trust, Manchester, United Kingdom; 8 Manchester Centre for Genomic Medicine, North West Genomic Laboratory Hub, Saint Mary’s Hospital, Manchester University NHS Foundation Trust, Manchester, United Kingdom; 9 Department of Gynaecological Surgery, The Christie NHS Foundation Trust, Manchester, United Kingdom; 10 Division of Evolution, Infection and Genomics, School of Biological Sciences, Faculty of Biology, Medicine and Health, The University of Manchester, Manchester, United Kingdom; 11 Department of Gynaecological Surgery, Manchester University NHS Foundation Trust, Manchester, United Kingdom; 12 Service d’oncologie médicale, Institut de Cancérologie des Hospices Civils de Lyon (IC-HCL), CITOHL, EPSILYON, Centre Hospitalier Lyon-Sud, Oullins-Pierre-Bénite, France

**Keywords:** Prognostic markers, Oncology

## Abstract

**Background:**

High-grade serous ovarian cancer (HGSOC) can be treated with platinum-based neoadjuvant chemotherapy (NACT) and delayed primary surgery (DPS). Histopathological response to NACT can be assessed using Böhm’s chemotherapy response score (CRS). We investigated whether germline *BRCA1/2* (g*BRCA1/2*) genotype associated with omental CRS phenotype.

**Methods:**

A retrospective study of patients with newly diagnosed FIGO stage IIIC/IV HGSOC prescribed NACT and tested for g*BRCA1/2* pathogenic variants (PVs) between September 2017 and December 2022 at The Christie Hospital. The Cox proportional hazards model evaluated the association between survival and key clinical factors. The chi-square test assessed the association between CRS3 (no/minimal residual tumour) and g*BRCA1/2* status.

**Results:**

Of 586 eligible patients, 393 underwent DPS and had a CRS reported. Independent prognostic factors by multivariable analysis were g*BRCA1/2* status (PV versus wild type [WT]), CRS (3 versus 1 + 2), surgical outcome (complete versus optimal/suboptimal) and first-line poly (ADP-ribose) polymerase-1/2 inhibitor maintenance therapy (yes versus no) (all *P* < 0.05). There was a non-significant trend for tumours with a g*BRCA2* PV having CRS3 versus WT (odds ratio [OR] = 2.13, 95% confidence intervals [CI] 0.95–4.91; *P* = 0.0647). By contrast, tumours with a g*BRCA1* PV were significantly less likely to have CRS3 than WT (OR = 0.35, 95%CI 0.14–0.91; *P* = 0.0291).

**Conclusions:**

Germline *BRCA1/2* genotype was not clearly associated with superior omental CRS. Further research is required to understand how HGSOC biology defines CRS.

## Background

Ovarian cancer is the second commonest cause of gynaecological cancer-related death worldwide, with more than 200,000 deaths attributed to the disease each year [1]. The most common histological subtype is high-grade serous ovarian cancer (HGSOC), which accounts for approximately 70% of all malignant tumours of the ovary [2]. The predilection of HGSOC to metastasise early to the peritoneal surfaces means most women are diagnosed with advanced-stage disease and cure is unlikely. Advanced HGSOC can be treated with neoadjuvant chemotherapy (NACT) followed by delayed primary surgery (DPS) [3–6]. A NACT plus DPS approach provides an opportunity to assess a tumour’s histopathological response to systemic anticancer therapy. However, there is no universally accepted scoring system to define the pathological response of HGSOC to NACT [7–14]. Moreover, the biology that defines histopathological responses to NACT in HGSOC is unknown. Consequently, all women treated with NACT plus DPS are offered the same platinum-containing chemotherapy in the immediate postoperative setting, potentially missing an opportunity to personalise therapy [15].

The genomic hallmarks of HGSOC are near-ubiquitous somatic *TP53* mutations, germline and somatic *BRCA1/2* mutations, homologous recombination deficiency (HRD) and genomic instability [16–20]. Germline *BRCA1/2* mutations occur in 10–20% of HGSOCs and are now routinely tested for in all newly diagnosed women [21]. Epithelial ovarian cancers harbouring a germline *BRCA1/2* mutation are highly sensitive to platinum-containing chemotherapy [22–25]. The putative mechanism for this platinum sensitivity is somatic loss (genetic or epigenetic) of the remaining *BRCA1/2* wild type allele, leading to a biallelic loss-of-function, a deficiency in homologous recombination, and an inability to repair lethal DNA double-strand breaks caused by platinum chemotherapy [26]. We hypothesised that because epithelial ovarian cancers containing a germline *BRCA1/2* mutation often have a better radiological response to platinum-containing chemotherapy compared to tumours with germline *BRCA1/2* wild type, they would also have a better histopathological response to NACT. To investigate this, we used the Böhm scoring system, which defines histopathological responses of HGSOC to NACT by examining omental tissue resected during DPS [12]. Tumours with a complete/near-complete response to NACT (i.e., no/minimal residual omental tumour) have a chemotherapy response score (CRS) of 3, while tumours with no/minimal response to NACT (i.e., viable residual omental tumour) have a CRS of 1 [12]. We hypothesised that HGSOCs harbouring a germline *BRCA1/2* mutation were more likely to have CRS3 than those with germline *BRCA1/2* wild type.

## Methods

### Patient cohort

A retrospective study in which eligible patients included those with newly diagnosed FIGO (International Federation of Gynaecology and Obstetrics) stage IIIC or IV [27] ovarian, fallopian tube or primary peritoneal cancer that were prescribed NACT at The Christie NHS Foundation Trust (Manchester, United Kingdom). All patients had histologically confirmed HGSOC and had been tested for germline *BRCA1/2* variants by the North West Genomic Laboratory Hub between 1^st^ September 2017 and 31^st^ December 2022. The decision to proceed to DPS after NACT was made by local multidisciplinary teams. All patients who received at least one dose of NACT were included in the overall population. All patients who underwent DPS were included in the DPS group.

### *BRCA1/2* testing

All patients provided informed consent for germline *BRCA1/2* testing. The next generation sequencing (NGS) and multiplex ligation-dependent probe amplification (MLPA) assays used in the North West Genomic Laboratory Hub have been reported previously [28]. Germline testing was performed on DNA extracted from peripheral blood lymphocytes. The NGS assay tested the coding regions of *BRCA1* and *BRCA2* plus 15 base pairs either side of each exon-intron boundary. Target coverage was at least 90% at a read depth of 100X. Small sequencing variants (<40 base pairs) were reported with a variant allele fraction ≥5%. Copy number variants including whole gene/exon deletions/duplications were detected using MLPA. Only pathogenic (class 5) and likely pathogenic (class 4) variants were reported (hereafter described as ‘pathogenic variants’ or ‘PVs’) [29].

### Efficacy outcomes

Chemotherapy response scores were determined by gynaecological Histopathologists by examining omental tissue removed at DPS using the Böhm scoring system [12]. Reporting Histopathologists were based at two tertiary referral centres, The Christie NHS Foundation Trust and Manchester University NHS Foundation Trust (Manchester, United Kingdom). The outcome of cytoreductive surgery was categorised as complete (no macroscopically visible tumour), optimal (1–10 mm of residual disease) or suboptimal (>10 mm of residual disease) [30]. Efficacy outcomes included progression-free survival (PFS), time to first subsequent therapy (TFST) and overall survival (OS). Progression-free survival was defined as the time interval from cycle 1 day 1 of NACT to the date of clinical or radiological progression or death, whichever occurred first. Time to first subsequent therapy was defined as the time interval from cycle 1 day 1 of NACT to the start of the subsequent therapy or death, whichever occurred first. Overall survival was defined as the time interval from cycle 1 day 1 of NACT to the date of death. The final date of data cut-off was 8^th^ January 2024 to allow a minimum of 12 months follow-up from cycle 1 day 1 of NACT in all patients. The GCIG CA 125 criteria was used to define response to NACT but not progression [31]. The GCIG CA 125 response was determined by comparing the pre-treatment serum CA 125 value with the pre-surgical serum CA 125 value. Only patients with a pre-treatment serum CA 125 value twice the upper limit of normal (range 0–30 U/mL) were eligible for GCIG CA 125 response assessment. The CA 125 ELIMination Rate Constant K (KELIM) was also used to define response to NACT [32]. It was calculated using the same model as implemented on the online calculator (https://www.biomarker-kinetics.org/) [33]. Only those patients with at least three CA 125 values measured within the first 100 days (or less) after starting NACT were eligible for KELIM.

### Statistical analysis

The chi-square test was used to determine whether there was an association between omental CRS3 (no/minimal residual tumour) and germline *BRCA1/2* status. Odds ratios (ORs) and corresponding 95% confidence intervals (95% CI) and *P* values were calculated. Kaplan–Meier statistics were used to estimate median survival times and corresponding 95% CI. Univariable and multivariable Cox proportional hazards regression was applied to evaluate the association between survival outcomes and key clinical factors in the overall population and DPS group. Hazard ratios (HRs) together with their corresponding 95% CI and *P* values were calculated. All reported *P* values were two-sided. Statistical analyses were performed with R version 4.3.1.

The study was approved by the Quality Improvement & Clinical Audit Committee at The Christie NHS Foundation Trust. The study was performed in line with the principles of the Declaration of Helsinki.

## Results

### Overall population

**Fig. 1:**
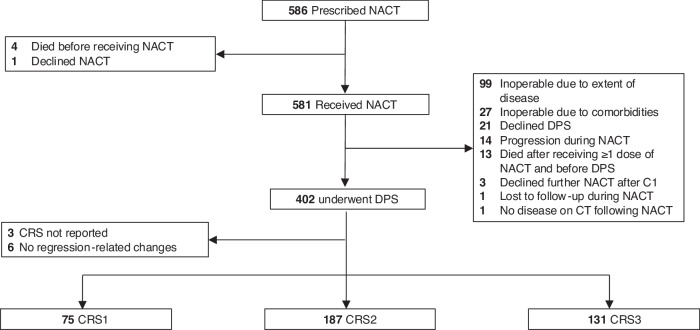
CONSORT diagram. Key: C1 cycle 1, CT computed tomography, CRS chemotherapy response score, DPS delayed primary surgery, NACT neoadjuvant chemotherapy.

In total, 586 eligible women were identified (Fig. 1). Of these, 581 patients received at least one dose of NACT and were included in the overall population (Supplementary Table S1). There were 77 (13%) germline *BRCA1/2* PVs detected (40 *BRCA1*, 37 *BRCA2*) in 77 women (Supplementary Data). The median PFS, TFST and OS for the overall population were 14.3 (95% CI 13.5–15.3; 474 events), 16.1 (95%CI 15.4–17.1; 462 events) and 35.7 months (95% CI 33.4–40.1; 337 events), respectively (Supplementary Fig. S1). The median follow-up for patients alive at the date of data cut-off was 30.1 months (interquartile range 19.8–34.9).

Univariable and multivariable analyses showed that ECOG performance status (0–1 versus 2–4), germline *BRCA1/2* status (PV versus wild type), cytoreductive surgery (yes versus no) and use of first-line poly (ADP-ribose) polymerase-1/2 inhibitor (PARPi) maintenance therapy (yes versus no) were prognostic factors in the overall population (Supplementary Tables S2 and S3). The median PFS was significantly longer in patients who received first-line maintenance therapy versus those who underwent surveillance (Supplementary Table S4). Taken together, these observations validated our overall population dataset and supported the next stage of evaluating omental CRS as a prognostic factor in women undergoing DPS.

### Delayed primary surgery group

Of the 581 patients who received at least one dose of NACT, 402 (69%) underwent DPS and 393 (98%) had an omental CRS reported (Supplementary Table S5). The 9 patients without a documented CRS had no evidence of neoplasia or chemotherapy-related regression changes in the omentum (*n* = 6) or no CRS reported (*n* = 3). Of the 179 patients who did not undergo DPS, the commonest reason for not undergoing debulking surgery was the extent of residual disease seen radiologically following NACT (*n* = 99 [55%]) (Fig. 1). Of these 99 patients, 57 (58%) had FIGO stage IV disease.

**Fig. 2:**
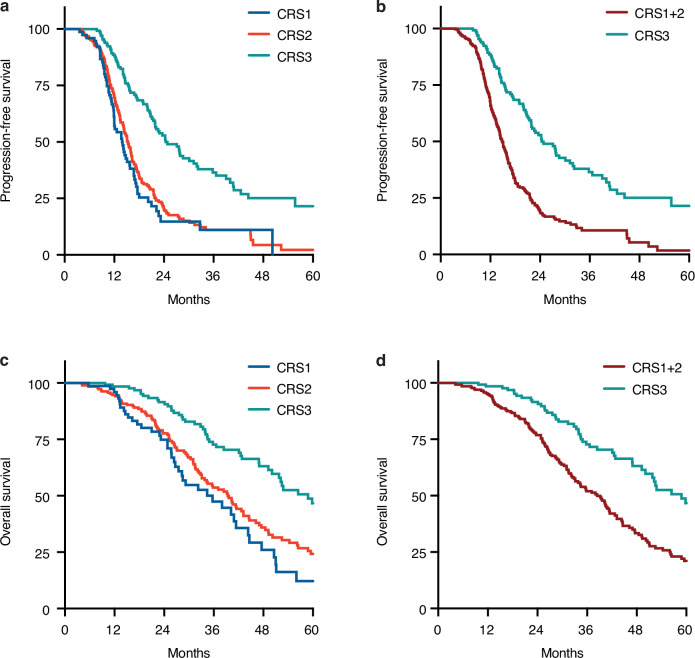
Kaplan–Meier curves for progression-free survival (PFS) and overall survival (OS) in CRS groups. **a**, **c** show Kaplan–Meier curves for PFS (**a**) and OS (**c**) for CRS1 versus CRS2 versus CRS3; **b**, **d** show Kaplan–Meier curves for PFS (**b**) and OS (**d**) for CRS1+2 versus CRS3.

Of the 393 patients in the DPS group with a CRS reported, 336 (85%) received 3–4 cycles of NACT and 246 (63%) had complete debulking (Table 1). The proportion of patients who received 3–4 cycles of NACT in the CRS3 versus CRS1 + 2 group did not differ significantly (114/131 [87%] versus 222/262 [85%], respectively; *P* = 0.60) (Table 1). Fifty-nine women received pre-operative bevacizumab (Table 1). There was no significant difference between the proportion of patients who received bevacizumab prior to DPS in the CRS3 versus CRS1 + 2 group (19/131 [15%] versus 40/262 [15%], respectively; *P* = 0.84) (Table 1). However, patients with CRS3 were more likely to achieve complete debulking than those with CRS1 + 2 (108/131 [82%] versus 138/262 [53%], respectively; *P* < 0.0001) (Table 1). Moreover, patients with CRS3 were more likely to have a GCIG CA 125 response (120/121 [99%] versus 229/246 [93%], respectively; *P* = 0.0085) or KELIM CA 125 favourable score (78/109 [72%] versus 112/218 [51%], respectively; *P* = 0.0005) than those with CRS1 + 2 (Table 2 and Supplementary Table S6). The median PFS, TFST and OS were significantly longer in the CRS3 group compared to the CRS1 + 2 group (Table 2, Fig. 2 and Supplementary Table S7).

**Table 1:** Demographic data for delayed primary surgery group.

	CRS1 + 2	CRS3
	*262 patients*	*131 patients*
Age at diagnosis/years		
Median (range)	63 (37–83)	67 (36–86)
ECOG performance status		
0–1	218 (83%)	109 (83%)
2–4	44 (17%)	22 (17%)
FIGO stage		
IIIC	180 (69%)	96 (73%)
IVA	30 (11%)	9 (7%)
IVB	52 (20%)	26 (20%)
Germline *BRCA1/2* status		
Pathogenic variant	40 (15%)	18 (14%)
Wild type	222 (85%)	113 (86%)
Neoadjuvant chemotherapy		
Carboplatin-Paclitaxel	256	126
3-weekly	202 (77%)	96 (73%)
Weekly	54 (21%)	30 (23%)
Carboplatin-Caelyx	4 (2%)	0
Carboplatin	2 (<1%)	5 (4%)
Cycles of neoadjuvant chemotherapy		
<3	1 (<1%)	0
3-4	222 (85%)	114 (87%)
>4	39 (15%)	17 (13%)
Median (range)	4 (2–6)	4 (3–6)
Pre-operative bevacizumab		
Yes	40 (15%)	19 (15%)
Median (range)	3 (1–6)	3 (2–4)
Hyperthermic Intraperitoneal Chemotherapy		
Yes	15 (6%)	6 (2%)
Surgical outcome		
Complete	138 (53%)	108 (82%)
Optimal	102 (39%)	20 (15%)
Suboptimal	22 (8%)	3 (2%)
Total cycles of first-line chemotherapy		
<6	17 (6%)	5 (4%)
≥6	245 (94%)	126 (96%)
Median (range)	6 (2–8)	6 (3–8)
First-line maintenance therapy		
None	73 (28%)	45 (34%)
Bevacizumab	90 (34%)	40 (31%)
Olaparib	15 (6%)	13 (10%)
Niraparib	52 (20%)	23 (18%)
Bevacizumab and olaparib	32 (12%)	10 (8%)

*DPS* delayed primary surgery, *ECOG* Eastern Cooperative Oncology Group, *FIGO* International Federation of Gynaecology and Obstetrics.

**Table 2:** Efficacy outcomes in delayed primary surgery group.

	CRS1 + 2	CRS3	Hazard ratio (95% CI)	*P* value
	*262 patients*	*131 patients*		
GCIG CA 125 response – number (%)				
Eligible	247 (94%)	121 (92%)	–	–
CA 125 Response	229 (93%)	120 (99%)	–	0.0085
No CA 125 response	18 (7%)	1 (<1%)	–	
CA 125 KELIM – number (%)				
Eligible	218 (83%)	109 (83%)	–	–
Favourable score (≥1.0)	112 (51%)	78 (72%)	–	0.0005
Unfavourable score (<1.0)	106 (49%)	31 (28%)	–	
Progression-free survival				
Events – number (%)	218 (83%)	80 (61%)	-	–
Median (95% CI) – months	14.8 (13.9–15.9)	24.4 (21.9–31.9)	2.45 (1.9–3.2)	<0.0001
Time to first subsequent therapy				
Events – number (%)	210 (80%)	80 (61%)	–	–
Median (95% CI) – months	16.4 (15.7–17.8)	26.81 (23.2–34.4)	2.42 (1.9–3.2)	<0.0001
Overall survival				
Events – number (%)	148 (56%)	47 (36%)	–	–
Median (95% CI) – months	38.0 (33.1–42.7)	58.8 (50.1–NR)	2.28 (1.6–3.2)	<0.0001

*95% CI* 95% confidence interval, *CRS* chemotherapy response score, *DPS* delayed primary surgery, *GCIG* Gynecologic Cancer Intergroup, *KELIM* ELIMination Rate Constant K.

Univariable and multivariable analyses showed that germline *BRCA1/2* status (PV versus wild type), surgical outcome (complete versus optimal/suboptimal debulking), CRS (3 versus 1 + 2) and use of first-line PARPi maintenance therapy (yes versus no) were prognostic factors in the DPS group (Table 3 and Supplementary Table S8). These observations validated our DPS group dataset and supported the next stage of evaluating the association between omental CRS3 and germline *BRCA1/2* status.

**Table 3:** Multivariable analysis of delayed primary surgery group.

	Progression-free survival			Time to first subsequent therapy			Overall survival		
	HR	95% CI	*P* value	HR	95% CI	*P* value	HR	95% CI	*P* value
Age at diagnosis	1.00	0.98–1.01	0.6925	1.00	0.98–1.01	0.6069	1.00	0.99–1.02	0.7802
ECOG performance status									
0–1 (Ref)	1.00	–	–	1.00	–	–	1.00	–	–
2–4	1.25	0.92–1.69	0.1603	1.22	0.89–1.67	0.2083	1.40	0.95–2.05	0.0858
FIGO stage									
IIIC (Ref)	1.00	–	–	1.00	–	–	1.00	–	–
IV	1.27	0.99–1.63	0.0572	1.27	0.99–1.64	0.0614	1.22	0.89–1.66	0.2161
Germline *BRCA1/2* status									
Pathogenic variant (Ref)	1.00	–	–	1.00	–	–	1.00	–	–
Wild type	1.44	1.01–2.07	0.0447	1.47	1.02–2.13	0.0398	1.70	1.08–2.68	0.0223
Number of cycles of NACT									
3–4 (Ref)	1.00	–	–	1.00	–	–	1.00	–	–
>4	0.84	0.60–1.18	0.3109	0.92	0.65–1.30	0.6351	0.91	0.60–1.38	0.6499
Surgical outcome									
Complete (Ref)	1.00	–	–	1.00	–	–	1.00	–	–
Optimal/suboptimal	1.74	1.35–2.23	<0.0001	1.79	1.39–2.31	<0.0001	1.60	1.18–2.17	0.0026
Chemotherapy response score									
3 (Ref)	1.00	–	–	1.00	–	–	1.00	–	–
1 + 2	2.34	1.77–3.09	<0.0001	2.27	1.71–3.01	<0.0001	2.08	1.45–2.97	0.0001
First–line PARPi therapy									
Yes (Ref)	1.00	–	–	1.00	–	–	1.00	–	–
No	2.35	1.79–3.09	<0.0001	2.37	1.79–3.14	<0.0001	1.64	1.07–2.51	0.0231

*95% CI* 95% confidence interval, *DPS* delayed primary surgery, *ECOG* Eastern Cooperative Oncology Group, *HR* hazard ratio, *NACT* neoadjuvant chemotherapy, *PARPi* poly (ADP-ribose) polymerase-1/2 inhibitor.

### Germline BRCA1/2 status and chemotherapy response score

**Fig. 3:**
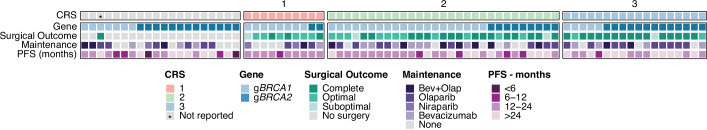
Chemotherapy response score reported for patients with a germline *BRCA1/2* pathogenic variant.

In the 393 patients in the DPS group with an omental CRS reported, there was no significant difference between the proportion of tumours with CRS3 in the germline *BRCA1/2* PV group versus the germline *BRCA1/2* wild type group (OR 0.88, 95% CI 0.49–1.63; *P* = 0.6875) (Table 4). When the subgroups of women with a germline *BRCA1* and *BRCA2* PV were analysed separately, there was a trend towards a significantly greater proportion of tumours with CRS3 in the germline *BRCA2* PV subgroup compared to the germline *BRCA1/2* wild type group (OR 2.13, 95% CI 0.95–4.91; *P* = 0.0647) (Table 4 and Fig. 3). In contrast, there was a significantly smaller proportion of tumours with CRS3 in the germline *BRCA1* PV subgroup compared to the germline *BRCA1/2* wild type group (OR 0.35, 95% CI 0.14–0.91; *P* = 0.0291) (Table 4 and Fig. 3).

**Table 4:** Association between chemotherapy response score and germline *BRCA1/2* (g*BRCA1/2*) status.

Group	CRS1 + 2	CRS3	Odds ratio (95% CI)	*P* value
Wild type	222 (66%)	113 (34%)	–	–
g*BRCA1*	28 (85%)	5 (15%)	0.35 (0.14–0.91)	0.0291
g*BRCA2*	12 (48%)	13 (52%)	2.13 (0.95–4.91)	0.0647
g*BRCA1/2*	40 (69%)	18 (31%)	0.88 (0.49–1.63)	0.6875

*95% CI* 95% confidence interval, *CRS* chemotherapy response score.

## Discussion

A key question in HGSOC research is what is the biology that drives histopathological responses to NACT. Improving our understanding of the biology that underpins responses to NACT may help optimise the use of postoperative therapies. At present, the standard approach for women with newly diagnosed, advanced-stage HGSOC, who are treated with NACT and DPS, is to offer the same platinum-based chemotherapy postoperatively that was used preoperatively, regardless of how well the tumour had responded histopathologically [15]. This treatment strategy misses a potential opportunity to develop personalised postoperative therapies through improved understanding of the biology that underpins a tumour’s histopathological response to NACT.

Epithelial ovarian cancers that harbour a germline *BRCA1/2* PV respond better radiologically to platinum-based chemotherapy compared to those with germline *BRCA1/*2 wild type [23]. Moreover, women with a germline *BRCA1/2* PV have longer survival outcomes than those with germline *BRCA1/*2 wild type, largely due to enhanced primary platinum sensitivity and retained platinum sensitivity in the relapsed setting [22, 24, 25]. In their original study, Böhm and colleagues reported that omental CRS3 had a negative predictive value of 94.6% for primary platinum resistance, implying that HGSOCs with CRS3 were highly unlikely to develop primary platinum-resistant disease [12]. Taken together, these observations suggest that one factor underpinning histopathological responses to NACT in HGSOC could be germline *BRCA1/2* status. Thus, we hypothesised that tumours with CRS3 were more likely to have a germline *BRCA1*/*2* PV compared to tumours with germline *BRCA1/2* wild type.

To evaluate an association between omental CRS phenotype and germline *BRCA1/2* genotype, we firstly confirmed that key clinical factors in our overall population and DPS group were prognostically reproducible. Here, we showed that undergoing cytoreductive surgery and achieving complete debulking were prognostic in our overall population and DPS group. Moreover, we showed that having CRS3 or a germline *BRCA1/2* PV were independent prognostic factors in our DPS group, regardless of the use of first-line PARPi maintenance therapy. These observations provided us with confidence that data from our cohort aligned with data reported in prospective ovarian cancer cohorts [30, 34–39]. Following this validation exercise, we were able to show a trend towards omental CRS3 in tumours harbouring a germline *BRCA2* PV compared to tumours with germline *BRCA1/2* wild type. In contrast, and surprisingly, we found that tumours with CRS3 were less likely to have a germline *BRCA1* PV than those with germline *BRCA1/2* wild type.

It is unclear why we found no clear association between germline *BRCA1/2* genotype and CRS phenotype. It is notable that in their original study, Böhm and colleagues did not use *BRCA1/2* status to develop the CRS, because germline *BRCA1/2* status was not known in most women included in their discovery or validation cohorts [12]. Thus, it is plausible that the histopathological changes seen in the omentum that define CRS, including the fibro-inflammatory response and extent of residual disease, are not biologically related to the presence of a germline *BRCA1*/*2* PV. A multicentre, individual patient data meta-analysis performed by The HGSC CRS Collaborative Network did report a positive associated between tumours with CRS3 and a germline *BRCA1/2* PV (*P* = 0.027) [40]. However, in this meta-analysis only 306 patients (35%) out of 807 included had known germline *BRCA1/2* status. Moreover, 80/306 (26%) had a germline *BRCA1/2* PV and 33/80 (41%) had CRS3. The prevalence of germline *BRCA1/2* PVs in this meta-analysis was higher than expected in an unselected population of women with ovarian cancer [41]. Thus, by unavoidably including a disproportionally high number of germline *BRCA1/2* heterozygotes, the meta-analysis may have overestimated the association between CRS3 and germline *BRCA1/2* status [40]. Nonetheless, the lack of a clear association between CRS3 and germline *BRCA1/2* status in our study, and other smaller observational studies [42–50], implies that CRS biology cannot be explained using the current dogma linking *BRCA1/2* status and platinum sensitivity in HGSOC. To deconvolute the complexity of CRS biology, detailed analysis of tumour-intrinsic factors and the tumour immune microenvironment is required, using spatial multiomic platforms and next-generation high-dimensional tissue imaging. Indeed, detailed understanding of the biology of the residual omental tumour and/or the tumour immune microenvironment in patients with CRS1 + 2 may lead to optimisation of post-operative treatments through personalised medicine.

What is evident from our study is that a large number of tumours with CRS3 have germline *BRCA1/2* wild type. These tumours may have a *BRCA1/2* wild type/HRD-positive genotype, although this remains unknown as routine genomic instability score (GIS) testing was not available during most of the study period [51]. Indeed, tumour HRD/GIS testing was only performed on 215/586 (37%) patients in the overall population (151/402 [38%] patients in the DPS group) because it only became available as a standard test in the North West of England in April 2021. Thus, further research is required to determine if omental CRS3 associates with tumour HRD/GIS status [52, 53]. Nevertheless, our study does validate CRS3 as an independent prognostic factor for primary platinum efficacy. It is well established that HRD-positive tumours are highly sensitive to PARPi, and that PARPi efficacy is linked to primary platinum response [34–39]. All women with newly diagnosed, advanced-stage HGSOC now undergo routine tumour HRD testing to access first-line olaparib-bevacizumab maintenance therapy [37]. Tumours with better histopathological responses to NACT are more likely to fail HRD testing due to the paucity of residual viable tumour cells available for testing in the DPS specimen [52]. In this scenario, when tumour HRD testing fails, omental CRS3 may offer a surrogate biomarker of PARPi efficacy. Thus, we advocate prospectively investigating omental CRS3 as a surrogate biomarker of PARPi efficacy; the main advantage of CRS being that it is cost-free [12].

Our retrospective study has some limitations based on the study design, which may have led to unavoidable selection bias. Firstly, germline *BRCA1/2* testing occurred over a five-year period, in which standard eligibility criteria for testing changed from relapsed, platinum-sensitive HGSOC to any patient diagnosed with HGSOC. As a result, not all women with newly diagnosed HGSOC that were treated at The Christie Hospital will have undergone germline *BRCA1/2* testing between 2017 and 2022. Indeed, patients who did not undergo germline *BRCA1/2* testing were not included in our study. Secondly, only those patients that underwent DPS could be included in our analysis of omental CRS phenotype versus germline *BRCA1/2* genotype, meaning the conclusions drawn about HGSOC biology cannot be applied to patients who did not receive NACT or undergo DPS. Thirdly, the decision to proceed with DPS was made by two multidisciplinary teams working in isolation, thus there may have been subtle differences between patients chosen for upfront primary surgery versus DPS. Fourthly, histology reporting occurred across two gynaecology pathology laboratories, so there may have been interobserver variation in CRS amongst reporting histopathologists, although this was not assessed in the study. One way to mitigate against interobserver variability would be to include a centralised review panel of expert gynaecology pathologists and/or use a digital pathology tool. Finally, we have not included patients with somatic *BRCA1/2* PVs in the analysis, largely because local testing showed that many of these mutations do not have variant allele frequencies consistent with biallelic loss-of-function [54]. Overall, however we were reassured by the fact that the Böhm scoring system was prognostic in our study, consistent with data reported by other observational studies [43, 45, 46, 48, 49, 55–64]. Moreover, median survival data in our overall population aligned with prospective trial data for patients with epithelial ovarian cancer treated with NACT [65].

To our knowledge, we report the largest observational study assessing the association between omental CRS and germline *BRCA1/2* status in newly diagnosed, advanced-stage HGSOC. Unexpectedly, we found no evidence to show a clear association between established pathology (omental CRS) and genetic (germline *BRCA1/2* status) biomarkers of platinum sensitivity in HGSOC. We also add to the growing evidence that the modified two-tier Böhm scoring system (CRS1 + 2 versus CRS3) is an independent prognostic biomarker in FIGO stage IIIC/IV HGSOC. Finally, we demonstrate that patients with CRS1 + 2 are less likely to have a GCIG CA 125 or KELIM CA 125 response to platinum-based NACT or achieve complete debulking surgery compared to those with CRS3.

## Supplementary information

Supplementary Table S1

Supplementary Table S2

Supplementary Table S3

Supplementary Table S4

Supplementary Table S5

Supplementary Table S6

Supplementary Table S7

Supplementary Table S8

Supplementary Figure S1

Supplementary Data

## Data Availability

The authors confirm that the data supporting the findings of this study are available within the article and its supplementary materials.
