# Supplementary material for: Germline BRCA1/2 status and chemotherapy response score in high-grade serous ovarian cancer
Source: Br J Cancer. 2024 Nov 16;131(12):1919–27. doi: 10.1038/s41416-024-02874-6 (PMC11628596; doi:10.1038/s41416-024-02874-6)
Supplement: Supplementary file 1 — Supplementary Table S1 [file 41416_2024_2874_MOESM1_ESM.docx]

**Supplementary Table S1. Demographic data for overall population.** Data is presented as number of patients (percent) unless otherwise stated. Key: ECOG, Eastern Cooperative Oncology Group; FIGO, International Federation of Gynaecology and Obstetrics.

|  | **Overall population** |
| --- | --- |
|  | *581 patients* |
| **Age at diagnosis – years**  Median (range) | 67 (36–89) |
| **ECOG performance status** |  |
| 0–1 | 440 (76%) |
| 2–4 | 141 (24%) |
| **FIGO stage** |  |
| IIIC | 374 (64%) |
| IVA | 58 (10%) |
| IVB | 149 (26%) |
| **Germline *BRCA1/2* status** |  |
| Pathogenic variant | 77 (13%) |
| Wild type | 504 (87%) |
| **Prescribed neoadjuvant chemotherapy** |  |
| Carboplatin-paclitaxel | 557 (96%) |
| 3-weekly | 395 (68%) |
| Weekly | 162 (28%) |
| Carboplatin-caelyx | 4 (1%) |
| Carboplatin | 20 (3%) |
| **Delayed primary surgery**  Yes | 402 (69%) |
| **Hyperthermic Intraperitoneal Chemotherapy**  Yes | 22 (4%) |
| **Total cycles of first-line chemotherapy**  Median (range) | 6 (1–8) |
| **Number of patients who received ≥6 cycles of first-line chemotherapy** | 516 (89%) |
| **First-line maintenance therapy** |  |
| None | 209 (36%) |
| Bevacizumab | 185 (32%) |
| Olaparib | 35 (6%) |
| Niraparib | 106 (18%) |
| Bevacizumab and olaparib | 46 (8%) |
