# Supplementary material for: Germline BRCA1/2 status and chemotherapy response score in high-grade serous ovarian cancer
Source: Br J Cancer. 2024 Nov 16;131(12):1919–27. doi: 10.1038/s41416-024-02874-6 (PMC11628596; doi:10.1038/s41416-024-02874-6)
Supplement: Supplementary file 2 — Supplementary Table S2 [file 41416_2024_2874_MOESM2_ESM.docx]

**Supplementary Table S2. Univariable analysis of overall population.** Key: 95% CI, 95% confidence interval; ECOG, Eastern Cooperative Oncology Group; FIGO, International Federation of Gynaecology and Obstetrics; HR, hazard ratio; PARPi, poly (ADP-ribose) polymerase-1/2 inhibitor.

|  | **Progression-free survival** | | | **Time to first subsequent therapy** | | | **Overall survival** | | |
| --- | --- | --- | --- | --- | --- | --- | --- | --- | --- |
|  | **HR** | **95% CI** | **P value** | **HR** | **95% CI** | **P value** | **HR** | **95% CI** | **P value** |
| **Age at diagnosis – years** | 1.01 | 1.00–1.02 | 0.0785 | 1.01 | 1.00–1.02 | 0.1732 | 1.01 | 1.00–1.03 | 0.0083 |
| **ECOG performance status** |  |  |  |  |  |  |  |  |  |
| 0–1 (Ref) | 1.00 | - | - | 1.00 | - | - | 1.00 | - | - |
| 2–4 | 1.69 | 1.37–2.08 | <0.001 | 1.67 | 1.36–2.07 | <0.0001 | 1.95 | 1.53–2.48 | <0.0001 |
| **FIGO stage** |  |  |  |  |  |  |  |  |  |
| IIIC (Ref) | 1.00 | - | - | 1.00 | - | - | 1.00 | - | - |
| IV | 1.42 | 1.18–1.71 | 0.0002 | 1.40 | 1.16–1.69 | 0.0005 | 1.33 | 1.07–1.66 | 0.0107 |
| **Germline *BRCA1/2* status** |  |  |  |  |  |  |  |  |  |
| Pathogenic variant (Ref) | 1.00 | - | - | 1.00 | - | - | 1.00 | - | - |
| Wild type | 1.91 | 1.43–2.55 | <0.0001 | 1.90 | 1.41–2.55 | <0.0001 | 1.73 | 1.23–2.42 | 0.0016 |
| **Cytoreductive surgery** |  |  |  |  |  |  |  |  |  |
| Yes (Ref) | 1.00 | - | - | 1.00 | - | - | 1.00 | - | - |
| No | 2.55 | 2.11–3.09 | <0.0001 | 2.47 | 2.04–2.99 | <0.0001 | 2.75 | 2.21–3.42 | <0.0001 |
| **First-line PARPi therapy** |  |  |  |  |  |  |  |  |  |
| Yes (Ref) | 1.00 | - | - | 1.00 | - | - | 1.00 | - | - |
| No | 2.36 | 1.90–2.93 | <0.0001 | 2.34 | 1.88–2.93 | <0.0001 | 1.86 | 1.37–2.54 | 0.0001 |
