# Supplementary material for: Germline BRCA1/2 status and chemotherapy response score in high-grade serous ovarian cancer
Source: Br J Cancer. 2024 Nov 16;131(12):1919–27. doi: 10.1038/s41416-024-02874-6 (PMC11628596; doi:10.1038/s41416-024-02874-6)
Supplement: Supplementary file 3 — Supplementary Table S3 [file 41416_2024_2874_MOESM3_ESM.docx]

**Supplementary Table S3.** **Multivariable analysis of overall population.** Key: 95% CI, 95% confidence interval; ECOG, Eastern Cooperative Oncology Group; FIGO, International Federation of Gynaecology and Obstetrics; HR, hazard ratio; PARPi, poly (ADP-ribose) polymerase-1/2 inhibitor.

|  | **Progression-free survival** | | | **Time to first subsequent therapy** | | | **Overall survival** | | |
| --- | --- | --- | --- | --- | --- | --- | --- | --- | --- |
|  | **HR** | **95% CI** | **P value** | **HR** | **95% CI** | **P value** | **HR** | **95% CI** | **P value** |
| **Age at diagnosis (years)** | 0.99 | 0.98–1.00 | 0.0807 | 0.99 | 0.98–1.00 | 0.0334 | 1.00 | 0.99–1.01 | 0.7672 |
| **ECOG performance status** |  |  |  |  |  |  |  |  |  |
| 0–1 (Ref) | 1.00 | - | - | 1.00 | - | - | 1.00 | - | - |
| 2–4 | 1.47 | 1.18–1.81 | 0.0004 | 1.46 | 1.18–1.82 | 0.0005 | 1.65 | 1.29–2.12 | 0.0001 |
| **FIGO stage** |  |  |  |  |  |  |  |  |  |
| IIIC (Ref) | 1.00 | - | - | 1.00 | - | - | 1.00 | - | - |
| IV | 1.08 | 0.89–1.31 | 0.4400 | 1.05 | 0.86–1.28 | 0.6376 | 1.07 | 0.85–1.35 | 0.5533 |
| **Germline *BRCA1/2* status** |  |  |  |  |  |  |  |  |  |
| Pathogenic variant (Ref) | 1.00 | - | - | 1.00 | - | - | 1.00 | - | - |
| Wild type | 1.74 | 1.29–2.35 | 0.0003 | 1.71 | 1.26–2.33 | 0.0007 | 1.45 | 1.02–2.07 | 0.0393 |
| **Cytoreductive surgery** |  |  |  |  |  |  |  |  |  |
| Yes (Ref) | 1.00 | - | - | 1.00 | - | - | 1.00 | - | - |
| No | 2.39 | 1.94–2.95 | <0.0001 | 2.32 | 1.88–2.87 | <0.0001 | 2.42 | 1.91–3.06 | <0.0001 |
| **First-line PARPi therapy** |  |  |  |  |  |  |  |  |  |
| Yes (Ref) | 1.00 | - | - | 1.00 | - | - | 1.00 | - | - |
| No | 2.03 | 1.62–2.53 | <0.0001 | 2.04 | 1.62–2.57 | <0.0001 | 1.65 | 1.20–2.26 | 0.0019 |
