# Supplementary material for: Germline BRCA1/2 status and chemotherapy response score in high-grade serous ovarian cancer
Source: Br J Cancer. 2024 Nov 16;131(12):1919–27. doi: 10.1038/s41416-024-02874-6 (PMC11628596; doi:10.1038/s41416-024-02874-6)
Supplement: Supplementary file 4 — Supplementary Table S4 [file 41416_2024_2874_MOESM4_ESM.docx]

**Supplementary Table S4. Progression-free survival in overall population according to first-line maintenance therapy.** Key: 95% CI, 95% confidence interval; HR, hazard ratio; NR, not reached.

| **First-line maintenance therapy** | **Progression-free survival** | | | | |
| --- | --- | --- | --- | --- | --- |
|  | **Patients** | **Events** | **Median (95% CI) / months** | **HR (95% CI)** | **P value** |
| None (Ref) | 208 | 193 | 11.02 (9.79–12.02) | 1.00 | - |
| Bevacizumab | 184 | 174 | 15.18 (14.00–16.66) | 0.74 (0.60–0.91) | 0.0047 |
| Niraparib | 106 | 75 | 14.42 (13.24–17.28) | 0.60 (0.46–0.79) | 0.0002 |
| Olaparib | 36 | 19 | 44.35 (23.56–NR) | 0.22 (0.14–0.35) | <0.0001 |
| Bevacizumab and olaparib | 46 | 13 | NR (NR–NR) | 0.15 (0.09–0.26) | <0.0001 |
