# Supplementary material for: Germline BRCA1/2 status and chemotherapy response score in high-grade serous ovarian cancer
Source: Br J Cancer. 2024 Nov 16;131(12):1919–27. doi: 10.1038/s41416-024-02874-6 (PMC11628596; doi:10.1038/s41416-024-02874-6)
Supplement: Supplementary file 5 — Supplementary Table S5 [file 41416_2024_2874_MOESM5_ESM.docx]

**Supplementary Table S5. Demographic data for delayed primary surgery group.** Data is presented as number of patients (percent) unless otherwise stated. Key: DPS, delayed primary surgery; ECOG, Eastern Cooperative Oncology Group; FIGO, International Federation of Gynaecology and Obstetrics.

|  | **DPS group** | **CRS1** | **CRS2** | **CRS3** | **CRS unknown** |
| --- | --- | --- | --- | --- | --- |
|  | *402 patients* | *75 patients* | *187 patients* | *131 patients* | *9 patients* |
| **Age at diagnosis – years**  Median (range) | 66 (36–86) | 64 (45–83) | 63 (37–81) | 67 (36–86) | 70 (54–84) |
| **ECOG performance status** |  |  |  |  |  |
| 0–1 | 334 (83%) | 64 (85%) | 154 (82%) | 109 (83%) | 7 (78%) |
| 2–4 | 68 (17%) | 11 (15%) | 33 (18%) | 22 (17%) | 2 (22%) |
| **FIGO stage** |  |  |  |  |  |
| IIIC | 282 (70%) | 51 (68%) | 129 (69%) | 96 (73%) | 6 (67%) |
| IVA | 39 (10%) | 5 (7%) | 25 (13%) | 9 (7%) | 0 |
| IVB | 81 (20%) | 19 (25%) | 33 (18%) | 26 (20%) | 3 (33%) |
| **Neoadjuvant chemotherapy** |  |  |  |  |  |
| 3-weekly carboplatin-paclitaxel | 304 (76%) | 64 (85%) | 138 (74%) | 96 (73%) | 6 (67%) |
| Weekly carboplatin-paclitaxel | 87 (22%) | 9 (12%) | 45 (24%) | 30 (23%) | 3 (33%) |
| Carboplatin-caelyx | 4 (1%) | 2 (3%) | 2 (1%) | 0 | 0 |
| Carboplatin | 7 (2%) | 0 | 2 (1%) | 5 (4%) | 0 |
| **Cycles of neoadjuvant chemotherapy** |  |  |  |  |  |
| <3 | 1 (<1%) | 0 | 1 (<1%) | 0 | 0 |
| 3–4 | 344 (86%) | 61 (81%) | 161 (86%) | 114 (87%) | 8 (89%) |
| >4 | 57 (14%) | 14 (19%) | 25 (13%) | 17 (13%) | 1 (11%) |
| Median (range) | 4 (2–6) | 3 (3–6) | 4 (2–6) | 4 (3–6) | 3 (3–6) |
| **Pre-operative bevacizumab**  Yes  Cycles – median (range) | 60 (15%)  3 (2–6) | 12 (16%)  4 (2–5) | 28 (15%)  3 (1–6) | 19 (15%)  3 (2–4) | 1 (11%)  3 |
| **Hyperthermic Intraperitoneal Chemotherapy**  Yes | 22 (5%) | 2 (3%) | 13 (7%) | 6 (2%) | 1 (11%) |
| **Surgical outcome** |  |  |  |  |  |
| Complete | 254 (63%) | 33 (44%) | 105 (56%) | 108 (82%) | 8 (89%) |
| Optimal | 123 (31%) | 33 (44%) | 69 (37%) | 20 (15%) | 1 (11%) |
| Suboptimal | 25 (6%) | 9 (12%) | 13 (7%) | 3 (2%) | 0 |
| **Total cycles of first-line chemotherapy** |  |  |  |  |  |
| <6 | 22 (5%) | 3 (4%) | 14 (7%) | 5 (4%) | 0 |
| ≥6 | 380 (95%) | 72 (96%) | 173 (93%) | 126 (96%) | 9 (100%) |
| Median (range) | 6 (2–8) | 6 (3–8) | 6 (2–8) | 6 (3–8) | 6 |
| **First-line maintenance therapy** |  |  |  |  |  |
| None | 120 (30%) | 20 (27%) | 53 (28%) | 45 (34%) | 2 (22%) |
| Bevacizumab | 135 (34%) | 26 (35%) | 64 (34%) | 40 (31%) | 5 (56%) |
| Olaparib | 29 (7%) | 4 (5%) | 11 (6%) | 13 (10%) | 1 (11%) |
| Niraparib | 76 (19%) | 15 (20%) | 37 (20%) | 23 (18%) | 1 (11%) |
| Bevacizumab and olaparib | 42 (10%) | 10 (13%) | 22 (12%) | 10 (8%) | 0 |
