# Supplementary material for: Germline BRCA1/2 status and chemotherapy response score in high-grade serous ovarian cancer
Source: Br J Cancer. 2024 Nov 16;131(12):1919–27. doi: 10.1038/s41416-024-02874-6 (PMC11628596; doi:10.1038/s41416-024-02874-6)
Supplement: Supplementary file 6 — Supplementary Table S6 [file 41416_2024_2874_MOESM6_ESM.docx]

**Supplementary Table S6.** **CA 125 response to neoadjuvant chemotherapy according to chemotherapy response score.** Key: CRS, chemotherapy response score; DPS, delayed primary surgery; GCIG, Gynecologic Cancer Intergroup; KELIM, ELIMination Rate Constant K.

|  | **DPS group** | **CRS1** | **CRS2** | **CRS3** | **CRS unknown** |
| --- | --- | --- | --- | --- | --- |
|  | *402 patients* | *75 patients* | *187 patients* | *131 patients* | *9 patients* |
| **GCIG CA 125 response** – number (%)  Eligible | 377 (94%) | 68 (91%) | 179 (96%) | 121 (92%) | 9 (100%) |
| GCIG CA 125 response | 358 (95%) | 61 (90%) | 168 (94%) | 120 (99%) | 9 (100%) |
| No GCIG CA 125 response | 19 (5%) | 7 (10%) | 11 (6%) | 1 (<1%) | 0 |
| **CA 125 KELIM** – number (%) |  |  |  |  |  |
| Eligible | 336 (84%) | 59 (79%) | 159 (85%) | 109 (83%) | 9 (100%) |
| Favorable score (≥1.0) | 195 (58%) | 22 (37%) | 90 (57%) | 78 (72%) | 5 (56%) |
| Unfavorable score (<1.0) | 141 (42%) | 37 (63%) | 69 (43%) | 31 (28%) | 4 (44%) |
