# Supplementary material for: Germline BRCA1/2 status and chemotherapy response score in high-grade serous ovarian cancer
Source: Br J Cancer. 2024 Nov 16;131(12):1919–27. doi: 10.1038/s41416-024-02874-6 (PMC11628596; doi:10.1038/s41416-024-02874-6)
Supplement: Supplementary file 7 — Supplementary Table S7 [file 41416_2024_2874_MOESM7_ESM.docx]

**Supplementary Table S7. Efficacy outcomes in each chemotherapy response score group.** Key: 95% CI, 95% confidence interval; CRS, chemotherapy response score; HR, hazard ratio; NR, not reached.

| **Efficacy outcome** | **CRS1** | **CRS2** | **CRS3** |
| --- | --- | --- | --- |
|  | *75 patients* | *187 patients* | *131 patients* |
| **Progression-free survival** |  |  |  |
| Events – number (%) | 60 (80) | 158 (84) | 80 (61) |
| Median (95% CI) / months | 13.9 (12.0–15.8) | 15.2 (14.2–16.30) | 24.4 (21.9–31.9) |
| **Time to first subsequent therapy**  Events – number (%)  Median (95% CI) / months | 59 (79)  15.3 (13.4–17.9) | 151 (81)  16.9 (16.1–19.0) | 80 (61)  26.8 (23.2–34.4) |
| **Overall survival** |  |  |  |
| Events – number (%) | 43 (57) | 105 (56) | 47 (36) |
| Median (95% CI) / months | 35.7 (27.5–44.6) | 39.4 (33.8–44.5) | 58.8 (50.0–NR) |
