# Supplementary material for: Germline BRCA1/2 status and chemotherapy response score in high-grade serous ovarian cancer
Source: Br J Cancer. 2024 Nov 16;131(12):1919–27. doi: 10.1038/s41416-024-02874-6 (PMC11628596; doi:10.1038/s41416-024-02874-6)
Supplement: Supplementary file 8 — Supplementary Table S8 [file 41416_2024_2874_MOESM8_ESM.docx]

**Supplementary Table S8. Univariable analysis of delayed primary surgery group.** Key: 95% CI, 95% confidence interval; DPS, delayed primary surgery; ECOG, Eastern Cooperative Oncology Group; FIGO, International Federation of Gynaecology and Obstetrics; HR, hazard ratio; NACT, neoadjuvant chemotherapy; PARPi, poly (ADP-ribose) polymerase-1/2 inhibitor.

|  | **Progression-free survival** | | | **Time to first subsequent therapy** | | | **Overall survival** | | |
| --- | --- | --- | --- | --- | --- | --- | --- | --- | --- |
|  | **HR** | **95% CI** | **P value** | **HR** | **95% CI** | **P value** | **HR** | **95% CI** | **P value** |
| **Age at diagnosis (years)** | 1.00 | 0.99–1.01 | 0.9226 | 1.00 | 0.99–1.01 | 0.9822 | 1.00 | 0.99–1.02 | 0.6892 |
| **ECOG performance status** |  |  |  |  |  |  |  |  |  |
| 0–1 (Ref) | 1.00 | - | - | 1.00 | - | - | 1.00 | - | - |
| 2–4 | 1.27 | 0.94–1.72 | 0.1229 | 1.26 | 0.92–1.72 | 0.1428 | 1.31 | 0.90–1.92 | 0.1615 |
| **FIGO stage** |  |  |  |  |  |  |  |  |  |
| IIIC (Ref) | 1.00 | - | - | 1.00 | - | - | 1.00 | - | - |
| IV | 1.37 | 1.08–1.75 | 0.0106 | 1.39 | 1.09–1.78 | 0.0086 | 1.28 | 0.94–1.73 | 0.1142 |
| **Germline *BRCA1/2* status** |  |  |  |  |  |  |  |  |  |
| Pathogenic variant (Ref) | 1.00 | - | - | 1.00 | - | - | 1.00 | - | - |
| Wild type | 1.57 | 1.12–2.20 | 0.0088 | 1.60 | 1.13–2.26 | 0.0077 | 1.69 | 1.11–2.58 | 0.0146 |
| **Number of cycles of NACT** |  |  |  |  |  |  |  |  |  |
| 3–4 (Ref) | 1.00 | - | - | 1.00 | - | - | 1.00 | - | - |
| >4 | 0.96 | 0.69–1.34 | 0.8087 | 1.00 | 0.71–1.40 | 0.9833 | 1.05 | 0.70–1.59 | 0.8076 |
| **Surgical outcome** |  |  |  |  |  |  |  |  |  |
| Complete (Ref) | 1.00 | - | - | 1.00 | - | - | 1.00 | - | - |
| Optimal/suboptimal | 2.16 | 1.71–2.74 | <0.0001 | 2.22 | 1.74–2.83 | <0.0001 | 1.95 | 1.47–2.59 | <0.0001 |
| **Chemotherapy response score** |  |  |  |  |  |  |  |  |  |
| 3 (Ref) | 1.00 | - | - | 1.00 | - | - | 1.00 | - | - |
| 1+2 | 2.45 | 1.88–3.18 | <0.0001 | 2.42 | 1.85–3.15 | <0.0001 | 2.28 | 1.64–3.18 | <0.0001 |
| **First-line PARPi therapy** |  |  |  |  |  |  |  |  |  |
| Yes (Ref) | 1.00 | - | - | 1.00 | - | - | 1.00 | - | - |
| No | 2.35 | 1.80–3.06 | <0.0001 | 2.36 | 1.80–3.10 | <0.0001 | 1.78 | 1.17–2.70 | 0.0073 |
