# Supplementary figures and images for: Germline BRCA1/2 status and chemotherapy response score in high-grade serous ovarian cancer
Source: Br J Cancer. 2024 Nov 16;131(12):1919–27. doi: 10.1038/s41416-024-02874-6 (PMC11628596; doi:10.1038/s41416-024-02874-6)

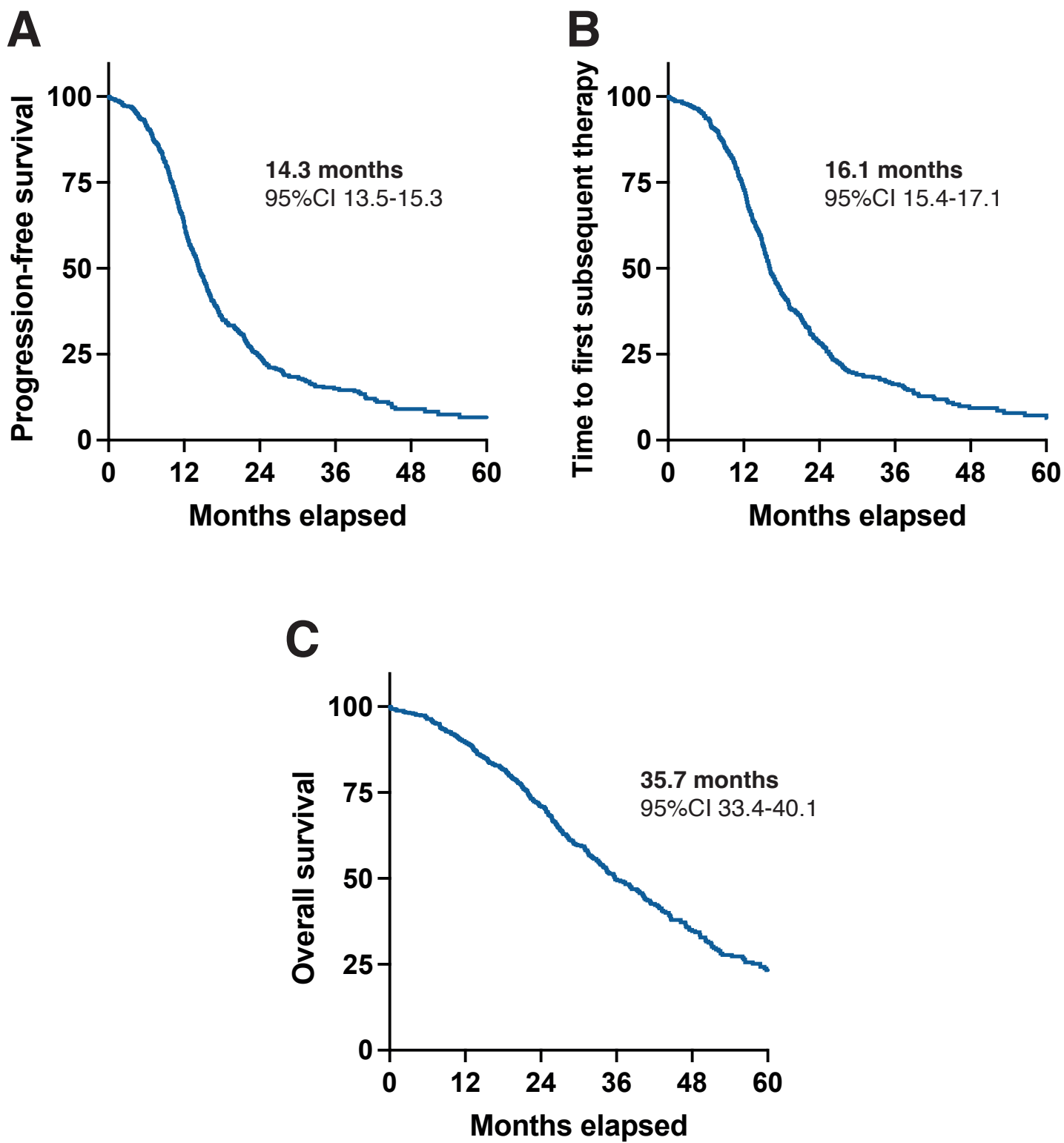

**Supplementary Figure S1**

Supplement: Supplementary file 9 — Supplementary Figure S1 [file 41416_2024_2874_MOESM9_ESM.pdf]
